# Activation of the peroxisome proliferator activated receptor γ counteracts sepsis-induced T-cell cytotoxicity towards alloantigenic target cells

**DOI:** 10.1186/cc14055

**Published:** 2014-12-03

**Authors:** A von Knethen, LK Sha, T Knape, L Kuchler, AK Giegerich, M Schulz, IA Hauser, B Brüne

**Affiliations:** 1 Institute of Biochemistry I - Pathobiochemistry, Faculty of Medicine, Goethe-University Frankfurt, Germany; 2 Fraunhofer Institute for Molecular Biology and Applied Ecology IME, Project Group Translational Medicine & Pharmacology TMP, Frankfurt am Main, Germany; 3 Department of Nephrology, Medical Clinic III, Frankfurt am Main, Germany

## Introduction

Sepsis originates from an uncontrolled inflammatory response. Despite intensive research, sepsis remains a major cause of death in ICUs. Therefore, new therapeutic approaches are mandatory. Taking into account that during sepsis progression cytotoxic T cells (CTL) are activated in an autoimmune fashion contributing to multiorgan damage, it remains unclear whether CTL are activated towards alloantigenic cells as well. This is especially important for patients receiving an immune suppressive therapy to permit organ transplantation and thus known to be at high risk for developing sepsis. Therefore, we analyzed whether sepsis activates CTL towards alloantigenic target cells and whether this can be inhibited by PPARγ activation, known to block T-helper cell responses.

## Methods

To characterize whether sepsis activates CTL and whether this can be inhibited by PPARγ activation, we used an *ex vivo *cytotoxicity assay to analyze CD8^+ ^T-cell-dependent cytotoxicity. Responder CD8^+ ^T cells were isolated from C57Bl/6N PPARγ wildtype (PPARγfl/fl) and T-cell specific knockout (Tc-PPARγ^-/-^) mice (haplotype H2Kb) following cecal ligation and puncture (CLP) versus sham treatment. P815 mastocytoma cells, a cell line originally derived from DBA/2 mice (haplotype H2Kd), were used as alloantigenic target cells. Pharmacological inhibition and/or activation of PPARγ *in vivo *and *ex vivo *was performed to clarify the impact of PPARγ in blocking CTL-dependent cytotoxicity. *In vivo*, PPARγ activity in wildtype mice was pharmacologically inhibited by the irreversible antagonist GW9662 or induced by the thiazolidinedione rosiglitazone. Systemic application of both compounds was performed intraperitoneally. A classic splenocyte-driven stimulation protocol to activate CTL was carried out as control.

## Results

CTL isolated from septic mice showed enhanced cytotoxicity towards alloantigenic P815 target cells. Enhanced cytotoxicity was effectively reduced by both PPARγ activation *in vivo *and *ex vivo*. In line, in CTL isolated from T-cell-specific PPARγ knockout (Tc-PPARγ^-/-^) mice PPARγ activation was ineffective, strengthening a PPARγ-dependent mechanism. At the molecular level *in vivo *and *ex vivo *activation of PPARγ reduced Fas and granzyme B expression in activated CTL, which might explain reduced cytotoxicity.

## Conclusion

Our study therefore suggests PPARγ activation *in vivo *to attenuate CTL-dependent alloantigenic cytotoxicity to possibly inhibit acute organ rejection.

